# AI-Assisted OCT Imaging for Core Needle Biopsy Guidance: The 1st in Humans Study

**DOI:** 10.3390/diagnostics16050811

**Published:** 2026-03-09

**Authors:** Nicusor Iftimia, Poonam Yadav, Michael Primrose, Gopi Maguluri, Jack Jones, John Grimble, Rahul Anil Sheth

**Affiliations:** 1 Physical Sciences Inc., Andover, MA 01810, USA; mprimrose@psicorp.com (M.P.); gmaguluri@psicorp.com (G.M.); jjones@psicorp.com (J.J.); jgrimble@psicorp.com (J.G.); 2 MD Anderson Cancer Center, Houston, TX 77030, USA; pyadav1@mdanderson.org (P.Y.); rasheth@mdanderson.org (R.A.S.)

**Keywords:** core needle biopsy, optical guidance, optical coherence tomography, artificial intelligence

## Abstract

**Background**: The heterogeneous nature of cancer with varying degrees of fat, necrosis, fibrosis, and varying degrees of tissue repair severely impacts the success of acquiring adequate tissue samples during percutaneous image-guided biopsy. Although ultrasound or CT fluoroscopy are used to identify tumor location and thus to guide biopsy needle insertion, these technologies do not provide the necessary resolution to determine tissue composition and enable the selection of the most appropriate location for biopsy specimen extraction. As a result, biopsy must be repeated, leading to significant cost to the health care system. **Methods**: In this study, we introduce a combined optical imaging/artificial intelligence (OI/AI) methodology for the real-time assessment of tissue morphology at the tip of the biopsy needle, prior to the collection of a biopsy specimen. Addressing a significant clinical challenge, this approach aims to reduce the proportion of biopsy cores—currently as high as 40%—that yield low diagnostic value due to elevated adipose or low tumor content. Our methodology employs micron-scale optical coherence tomography (OCT) imaging to obtain detailed structural tissue information using a minimally invasive needle probe. The OCT images are automatically analyzed using a convolutional neural network (CNN)-driven AI software developed by our team. A U-net style architecture was used to segment regions of tumor from the OCT scans. U-Net is a specialized convolutional neural network (CNN) architecture designed for fast, precise image segmentation, which involves classifying each pixel in an image to outline objects. This streamlined approach shows promise to provide clinicians with real-time results, supporting more accurate and informed decisions regarding biopsy site selection. To evaluate this technology, we conducted a clinical study using a custom-made OCT imager and recorded OCT images from patients diagnosed with liver cancers. Expert OCT interpreters supplied annotated reference images that were used to train a custom AI algorithm. **Results**: OCT imaging with ~10 mm axial and 20 mm lateral resolution enabled the collection of high-quality images of the tissue. The AI analysis was performed offline. UNet achieved an AUC of ~0.877 on the validation dataset, indicating promising performance for the relatively small data set used to train the model. The AI model matched human interpretations approximately 90% of the time, highlighting its promise for making biopsy procedures both more accurate and more efficient. **Conclusions**: A novel OCT instrument and AI software were evaluated for assessing tissue composition at the tip of biopsy needle. The OCT instrument produced micron-scale resolution images of the tissue, enabling AI analysis and accurate real-time discrimination of tissue type. This preliminary study demonstrated the clinical potential of this technology for improving biopsy success.

## 1. Introduction

Real-time evaluation of tissue cellularity at the biopsy needle tip has the potential to significantly improve the outcome of core needle biopsies. In this context, cellularity refers to the ratio of tumor tissue to benign or stromal components. The heterogeneous characteristics of tissue in certain patients, such as varying degrees of necrosis, fibrosis, and tissue repair, pose challenges to obtaining sufficient tissue samples during percutaneous image-guided biopsy procedures [1]. Due to this heterogeneity, the sensitivity and specificity of biopsies can vary considerably, typically ranging from 65% to 95% [2,3,4,5]. While ultrasound or CT fluoroscopy are routinely used to localize tumors and guide needle placement, these imaging modalities lack the resolution required for accurate assessment of tissue cellularity. Consequently, biopsies are often repeated, resulting in increased healthcare costs [6,7,8]. Additionally, inadequate specimen quality may impede clinicians’ ability to determine the most appropriate oncologic treatment strategy [9].

To address the evolving requirements for improved biopsy sampling adequacy, several optical techniques have been investigated, including Raman spectroscopy, dynamic light scattering, and optical coherence tomography (OCT) [10,11,12,13]. Of these, OCT demonstrates significant potential owing to its capability to accurately evaluate tissue morphology and facilitate rapid imaging of larger tissue volumes compared to alternative modalities. OCT has already been implemented for distinguishing normal tissue from cancerous tissue in multiple organs [14,15,16,17,18,19,20,21]. Nonetheless, commercially available OCT imaging systems remain financially restrictive, while appropriate OCT probes for transcutaneous in situ tissue examination are currently unavailable. These probes must satisfy both image quality and safety standards to minimize the risk of inadvertent tissue disruption during scanning.

To address these challenges, our team has developed a lower speed/lower cost OCT imaging approach and a specially designed fiber optic probe, which does not pose any risk for tissue disruption, as it scans insides of the biopsy needle. This probe can be safely placed within most 16 Ga to 20 Ga biopsy guidance needles, enabling accurate assessment of tissue cellularity prior to biopsy collection. The acquired OCT images are processed and rendered as two-dimensional maps, depicting tissue morphology at micron-scale resolution. The morphological maps are subsequently analyzed using a machine learning algorithm to quantify tissue cellularity. The resulting information is presented to the interventional radiologist as a pie or bar chart indicating tissue composition at the tip of the biopsy needle.

Building on prior advancements of the technology [22], this ongoing work focuses on further refining both the OCT instrument and the imaging probe, as well as on AI algorithms to enhance tissue characterization and biopsy guidance in clinical workflows. We demonstrate a clinic-ready instrument and probe, along with a preliminary framework for real-time image processing and interpretation. By integrating real-time feedback from high-resolution OCT imaging with robust machine learning interpretation, clinicians may be able to make informed decisions at the point of care, especially in challenging cases with complex tissue heterogeneity. Ultimately, this approach aims to streamline biopsy procedures, reduce non-diagnostic sampling, and improve outcomes for patients undergoing image-guided interventions, particularly those with hepatic malignancies, where accurate tissue sampling is critical for effective treatment planning.

## 2. Materials and Methods

**Figure 1:**
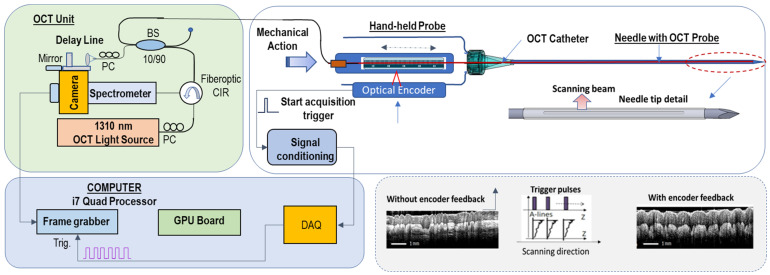
OCT imaging scheme based on encoder triggered A-line acquisition.

OCT Instrumentation: A novel OCT imaging approach (see schematic in Figure 1), previously described [23], has been adopted for this study. Using this approach, therecording of OCT data is based on feedback from an optical encoder that monitors the movement of the imaging probe, and thus it supports the use of a simple scanning engine, consisting of a linear motor. Briefly, a tissue reflectivity profile (A-line) is acquired whenever the linear encoder detects any movement of the OCT catheter along the central axis of the probe. Each encoder signal triggers a data acquisition (DAQ) card to start OCT data acquisition and processing. Every acquired tissue reflectivity profile (OCT A-scan) is processed (Fast Fourier Transformed, FFT) and added to an array to form an OCT image. This approach enables the acquisition of large-field-of-view OCT images at a moderate speed (1–2 fps), suitable for biopsy guidance.

The developed OCT instrument is based on the spectral-domain approach and uses a 1310 nm super-luminescent diode (SLD) light source with a bandwidth of approximately 85 nm, providing an axial resolution of ~10 μm (measured at Full Width at Half Maximum (FWHM)), which supports the detection of tissue features at the cellular level. The light from the broadband source is split into the sample and reference arms of the interferometer by a 10/90 fiber splitter. A fiber optic circulator is inserted between the light source and fiber splitter to maximize the return from both arms of the fiber interferometer. The light returned from the sample arm is mixed with that from the reference arm and sent to a spectrometer equipped with a linear array camera (Sensors Unlimited, Princeton, NJ, USA) with 20,248 pixels. When the two arms of the interferometer are matched within the coherence length of the light source, an interference pattern is produced, with fringes of different frequencies, depending on tissue structure. The fringe signals are digitized by a camera link frame grabber and processed in real-time by a Graphical Processing Unit (GPU), model GeForce RTX 3060 (Amazon Inc., Seattle, WC, USA). This powerful GPU is mainly needed for AI data analysis.

**Figure 2:**
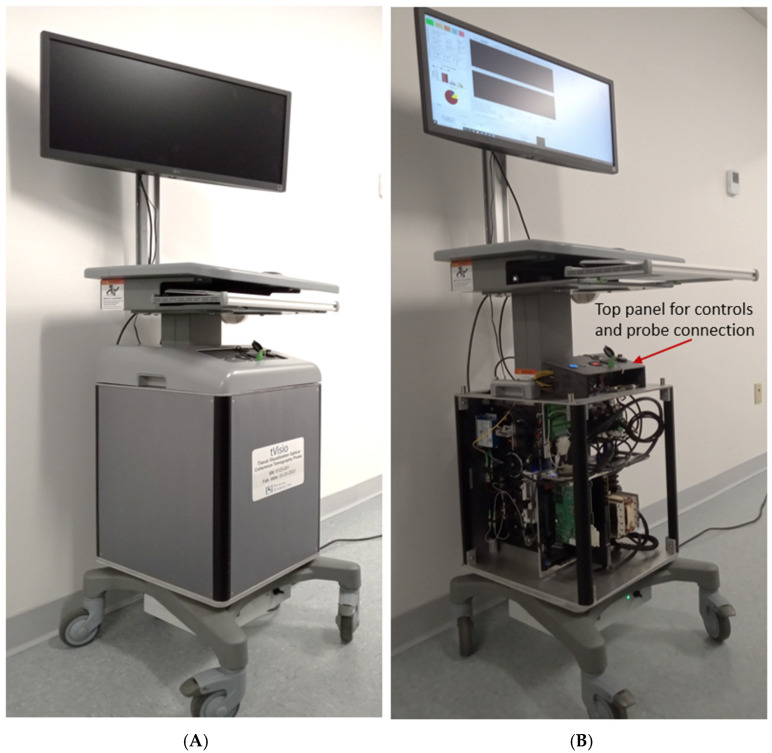
Photographs of the OCT instrumentation unit and probes: (**A**)—Fully assembled OCT instrument; (**B**)—OCT instrument with the covers removed, showing the interior details.

Photographs of the OCT instrumentation unit are shown in Figure 2. As observed, the OCT unit is placed on a wheeled cart, which can be easily rolled in and out of the clinical room. The instrumentation cart includes a computer equipped with the frame grabber and DAQ cards (National Instruments, Austin, TX, USA) for data acquisition and processing, as well as with an NVIDIA Graphical Processing Unit (GPU) for real-time processing of the OCT data, a power supply, and controllers for the hand-held probe motors.

As may be observed, our design provides easy access to hardware, enabling easy servicing of the device. Communication with the probe is made through electrical and fiber optic cables connected to the top panel of the instrumentation unit. This panel also includes buttons for computer start, a handheld probe power supply, and USB and network slots.

**Figure 3:**
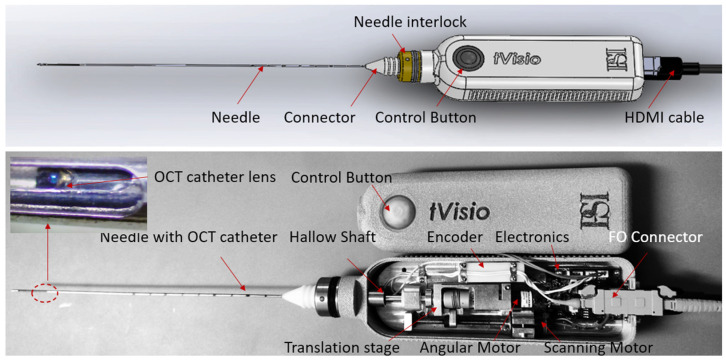
Biopsy probe CAD design and implementation. (**Top**)—general view; (**Bottom**)—assembled probe showing scanning mechanism details.

OCT probe: A handheld OCT probe, designed for use in a clinical setting, was developed. The CAD schematic and a photograph of the fully assembled probe are shown in Figure 3. The probe comprises several key components: the main body, an axial translation stage, a rotary orientation mechanism, an encoder, an electronic board, and a needle housing the OCT fiber optic catheter. The needle is only used to enable OCT probe advancement in the tissue, beyond the tip of the guidance needle, and not to collect tissue.

The OCT catheter is attached to the scanning engine via a fiber-optic connector, placed within a hollow shaft, mechanically connected to a combined axial/rotary motion engine. The axial motion is enabled by a linear motor. The rotary motion, enabled by a 2nd motor, is used to provide correct angular positioning of the OCT catheter relative to the slit practiced within the needle (20 mm long and 350 μm wide). The angular alignment is performed only once, when the probe is initialized, before the procedure, as a step of the instrument QA process. Upon instrument initialization, the motor incrementally rotates the catheter (2–5 degrees per step), while the data processing unit monitors OCT fringe amplitude. Improper alignment results in excessive reflection from the needle walls, saturating the OCT spectrometer camera. Proper alignment allows the OCT light to exit through the designated slit at the needle tip. After analyzing the signal at each angular position, the shaft is finally oriented in the angular position that provides the minimum reflectance. The slot region of the needle is enclosed by a fluorinated ethylene propylene (FEP) tube, which seals the OCT catheter within the needle and prevents tissue entrapment during operation. To secure the FEP tube in place, the needle is slightly tapered towards its tip, where the axial slot is located.

The translation stage only operates when the user pushes the probe control button. An optical scale attached to the stage, oriented towards an optical encoder, produces a trigger pulse for every 5 μm increment of movement. Accordingly, a 10 mm scan generates 2000 pulses, enabling the acquisition of an equivalent number of A-scans that are subsequently combined to construct an image. An electronic circuit placed into the probe body is used to enable an A-line acquisition only when the catheter is moved intentionally, after pressing the control button. Thus, it blocks false triggers generated by the small vibrations during probe insertion within the guidance needle, before pressing the button. This circuit is also formatting the trigger signal, so it can be reliably sent through a 2 m length mini-HDMI cable to the instrumentation unit to start data acquisition.

The custom-made needle is attached to the probe holder via a luer-lock cap, similar to that of commercial syringes. Thus, this needle can be easily replaced during the procedure, if needed.

The OCT fiber catheter consists of a single-mode (SM) fiber, terminated with a micro lens polished at 45 deg to send the light orthogonal to the catheter scanning direction. The catheter is encapsulated within a 460 um outer diameter hypodermic tube, terminated with a fiber-optic connector (Model DMI, Diamond USA, North Billerica, MA, USA).

**Figure 4:**
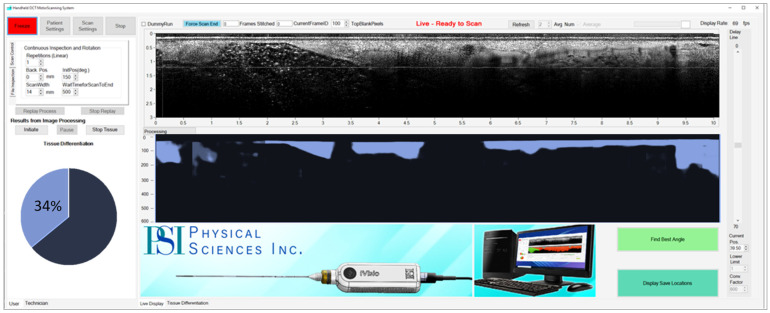
OCT instrument Graphical User Interface. (**Top**) Recorded image; (**Bottom**) Ai-segmented image showing cancer presence (in blue).

Instrument data acquisition and processing software: The software was designed to provide easy instrument setup, to control data acquisition and processing, and to save OCT cross-sectional images in real time. A screenshot of the Graphical User Interface (GUI) is presented in Figure 4.

The software was written in C++ for robustness and divided into 4 modules: one for loading patient information (4-digit number), one for probe motors control, one for data acquisition and processing, and one for data saving. These modules are controlled by associated buttons on the GUI. The GUI also includes windows for visualizing the data-saving path and settings of the motor’s parameters. The acquired cross-sectional images are displayed at the end of each axial scan of the probe. After acquiring each OCT image, the PSI custom-developed AI software analyzes and displays tissue composition.

**Data collection:** A human in vivo study was conducted at MD Anderson Cancer Center (MDACC), Houston, TX, USA, to preliminarily evaluate this technology. All experiments were performed in accordance with the MDACC IRB-approved protocol—No. 2023-0117. The scope of the study was to collect OCT images, correlate them with histopathology results, and train an AI algorithm to assess its capability for automatically assessing tissue cellularity. Over 35 patients with radiologically identified liver masses, scheduled for regular biopsy, were consented for this study. However, in the end, 25 patients were enrolled, as some declined to participate or were not eligible for the study (had co-morbid conditions that did not allow for additional procedures). A biopsy guidance needle was first placed under US guidance within the radiologically identified areas as being suspicious of cancer. Then, the stylet of this needle was removed, and the OCT needle was inserted, while constantly monitoring the marks on the needle, to ensure correct depth insertion. Multiple OCT scans were collected at each biopsy site using four angular orientations of the probe—0 deg, 90 deg, 180 deg, and 270 deg—relative to the insertion point. The regular biopsy procedure was then performed. The extracted biopsy specimens were collected from the same angular orientations of the notch of the biopsy needle and labeled to show the quadrant. OCT needle and biopsy needle depth insertion were carefully monitored and kept within 1 mm precision by monitoring the marks on each needle relative to the biopsy guidance needle entrance point. However, small registration errors can still occur in this process.

OCT results were not used to guide biopsy, as this was not a clinical trial to assess the effectiveness of OCT guidance. All procedures went smoothly, without any complications, and the OCT imaging part did not extend the duration of the procedure by more than 5 min.

## 3. AI Training and Processing Results

The collected OCT images were correlated with the histopathology of the collected biopsy specimens and used to train the AI algorithm. Over 100 images corresponding to each tissue type (healthy and tumor) were selected for the AI algorithm training set. These images were selected in collaboration with the pathologist, to best match the pathology findings of the collected specimens. Furthermore, the selected images were further divided into smaller patches, increasing the training set size by a factor of 10.

**Figure 5:**
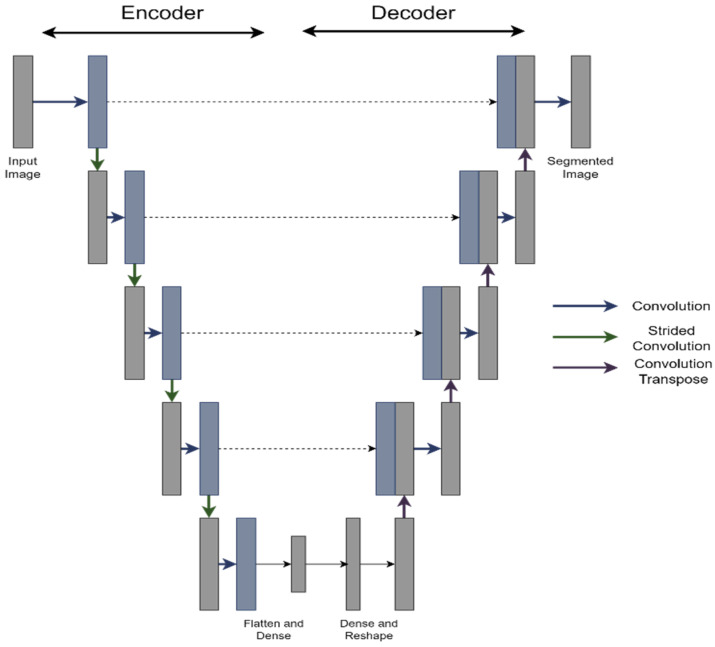
U-Net block diagram.

A specialized U-net-inspired artificial intelligence architecture was developed and implemented to segment tumor regions from OCT scans. The corresponding custom U-net architecture block diagram is depicted in Figure 5. In summary, feature encoding was performed using alternating convolutions with and without stride, and encoded features were integrated via two dense connections. Subsequently, the final dense layer underwent reshaping and up-sampling via alternating transposed convolutions and convolutional layers, culminating in a final convolution producing the segmented output.

Table 1 provides an overview of the number of filters and dense-layer nodes utilized. Strided convolutions employed filters consistent with those used in the preceding convolutional layer. To minimize model size—thereby reducing overfitting risk and enhancing processing speed—the number of filters per layer was deliberately limited compared to typical models. For training, images were randomly sampled from the training set, and a random horizontal window of 400 pixels (covering the full vertical range) was selected; this window was then resized to 256 × 256 for input into the U-net. Training employed mean-squared error as the loss function and optimization was performed using Adaptive Moment Estimation (Adam). This streamlined approach was selected over alternative state-of-the-art methods due to time constraints and to illustrate the efficacy of a lightweight segmentation model trained exclusively on available OCT data.

After algorithm training, another set of OCT scans was processed using the UNet trained model and compared against the OCT reader annotated images.

**Figure 6:**
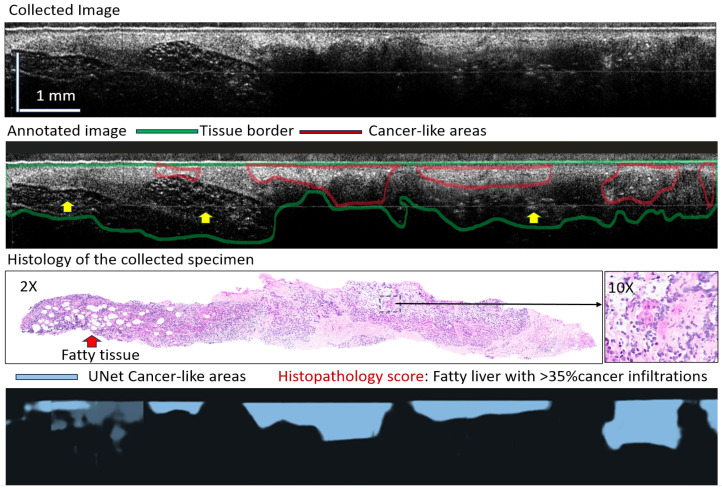
Representative example of AI findings for a heterogeneous tissue. Yellow arrows in the annotated image indicate areas of fatty tissue. Dark-blue areas in the segmented bottom image indicate algorithm uncertainty of cancer presence.

**Figure 7:**
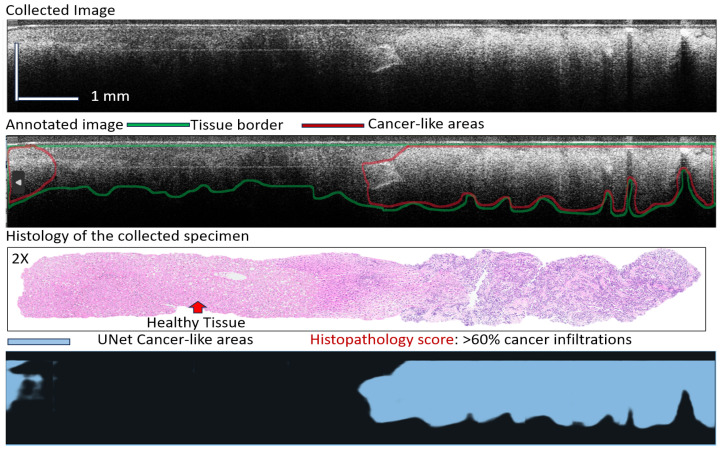
Representative example of AI findings for a liver tissue with clear invasive cancer.

Figure 6 and Figure 7 show two representative cases of tissue type differentiation using the AI algorithm. The first case (see Figure 6) is that of a highly heterogeneous tissue location: fatty liver with cancer infiltrations. The AI findings were in relatively good correlation with those of the annotators and especially with the histopathology of the collected biopsy core.

As observed, the cancerous areas (marked in blue) were reasonably differentiated. These areas are characterized by increased scattering due to the presence of cancer cells with large nuclei. However, since cancer had only partially invaded healthy tissue, it was a challenging task for both the annotator and the AI to precisely demarcate the margins, as morphology changes were not abrupt (see dark blue areas in Figure 6).

The 2nd case is that of better differentiated cancer tissue, where the cancer cells have fully invaded a large area of the liver tissue (see the red-annotated areas in Figure 7, which were confirmed by AI). It is to be noted that OCT penetration is lower in the relatively healthy areas of the liver, neighboring cancer infiltrations. This might be explained by the high-water content in patients with signs of cirrhosis, where fluid builds up in the abdomen, causing a swollen belly, weight gain, and discomfort. In healthy patients, the penetration of the OCT light is much higher, as it is not diminished by water absorption.

**Figure 8:**
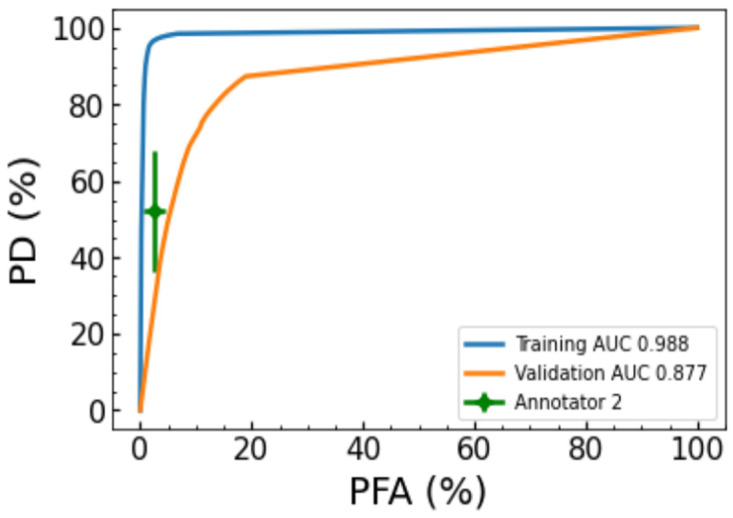
ROC curves comparing the tumor PD and PFA for the training data (blue), withheld validation data (orange) and a single point showing the average PD/PFA with one standard deviation across images comparing annotator 1 and annotator 2. The UNet was trained using Annotator 1.

To benchmark the preliminary performance of the UNet for the tumor segmentation task, the tumor probability of detection (PD) and tumor probability of false alarm rate (PFA) were calculated on a per-pixel basis across all training and validation images. Figure 8 shows receiver operating characteristic curves for the per-pixel tumor detection rates across all training images (blue) and validation images (orange). On the training data, the UNet achieved an area under the curve (AUC) of ~0.988, indicating high capability in reproducing the annotated regions. The UNet also achieved an AUC of ~0.877 on the validation dataset, indicating decent performance on the dataset.

## 4. Discussion

Study Overview: This paper presents the first in-human study of transcutaneous optical coherence tomography (OCT) imaging to visualize tissue morphology at the tip of the biopsy needle and assess tissue cellularity. The primary objective was to evaluate the technology’s ability to capture high-quality images and to determine the effectiveness of an artificial intelligence (AI) model in automatically analyzing these images and estimate the percentage of tumor tissue within the targeted area. For this purpose, a minimally invasive OCT probe and a custom instrument were designed and used for the reported study.

AI Model Performance and Limitations: The AI model developed for image analysis demonstrated a strong correlation with human annotations of the OCT images. However, discrepancies were observed in certain regions where the model was unable to accurately distinguish between tumor and partially infiltrating tumor areas. These limitations in model accuracy are attributed to two interconnected factors: challenges in annotation and model training. First, some tissue regions are inherently difficult for human annotators to identify and annotate with high accuracy, which is essential for effective AI model training and optimization. If annotators cannot establish clear ground truth for areas with overlapping morphologies in OCT images, the AI model cannot be expected to outperform human recognition. In essence, if humans are unable to differentiate subtle visual patterns—such as varying shades of white, gray, and black—and assign them to specific classes like “tumor” or “non-tumor,” the model will face the same challenge.

Improving Classification Accuracy: To enhance the classification accuracy for morphologically overlapping classes, it will be beneficial to increase the number of images that clearly represent challenging morphologies. Additionally, the inclusion of more examples of low-frequency classes (admixed fat–normal–tumor) can contribute to model performance improvement. In this study, agreement at the class level for regions of admixed tumor was infrequent and resulted in the lowest accuracy scores.

## 5. Conclusions

A novel AI-OCT method was evaluated for assessing tissue composition at the tip of a biopsy needle. OCT produced high-resolution images of the tissue interface and AI analysis of these images showed promising potential for real-time tissue characterization. Nevertheless, further development is needed to enhance the technology’s accuracy and support broader clinical implementation. Expanding the training dataset with larger image sets is considered essential. A continuation human study is expected to yield more comprehensive image datasets, which will improve AI performance.

## Figures and Tables

**Table 1:** The number of filters for convolutional layers and the number of nodes for the dense connections.

Layer	Num Filters [Num Nodes]
Conv 1	16
Conv 2	32
Conv 3	64
Conv 4	128
Conv 5	256
Dense 1	[512]
Dense 2	[16,384]

## Data Availability

The patient data cannot be shared; signal processing algorithms can be shared based on a business agreement.
